# Stability and resilience of the intestinal microbiota in children in daycare – a 12 month cohort study

**DOI:** 10.1186/s12866-018-1367-5

**Published:** 2018-12-22

**Authors:** Martin Steen Mortensen, Betina Hebbelstrup Jensen, Jeanne Williams, Asker Daniel Brejnrod, Lee O’Brien Andersen, Dennis Röser, Bente Utoft Andreassen, Andreas Munk Petersen, Christen Rune Stensvold, Søren Johannes Sørensen, Karen Angeliki Krogfelt

**Affiliations:** 1 Department of Bacteria, Parasites and Fungi, Statens Serum Institut, DK2300, Copenhagen S, Artillerivej 5 Denmark; 2 Department of Biology, Section of Microbiology, Copenhagen University, Copenhagen, Denmark; 3 Department of Pediatrics, Hvidovre Hospital, Copenhagen, Denmark; 4 Department of Pediatrics, H.C. Andersen’s Hospital, Odense, Denmark; 5 Department of Clinical Microbiology, Hvidovre Hospital, Copenhagen, Denmark; 6 Department of Gastroenterology, Hvidovre Hospital, Copenhagen, Denmark; 7 Department of Internal Medicine, Amager Hospital, Copenhagen, Denmark

**Keywords:** Gut microbiota, Preschool microbiota, Microbiota stability

## Abstract

**Background:**

We performed a 12-month cohort study of the stability and resilience of the intestinal microbiota of healthy children in daycare in Denmark in relation to diarrheal events and exposure to known risk factors for gastrointestinal health such as travelling and antibiotic use. In addition, we analyzed how gut microbiota recover from such exposures.

**Results:**

We monitored 32 children in daycare aged 1–6 years. Fecal samples were submitted every second month during a one-year observational period. Information regarding exposures and diarrheal episodes was obtained through questionnaires. Bacterial communities were identified using 16S rRNA gene sequencing. The core microbiota (mean abundance > 95%) dominated the intestinal microbiota, and none of the tested exposures (diarrheal events, travel, antibiotic use) were associated with decreases in the relative abundance of the core microbiota. Samples exhibited lower intra-individual variation than inter-individual variation. Half of all the variation between samples was explained by which child a sample originated from. Age explained 7.6–9.6% of the variation, while traveling, diarrheal events, and antibiotic use explained minor parts of the beta diversity. We found an age-dependent increase of alpha diversity in children aged 1–3 years, and while diarrheal events caused a decrease in alpha diversity, a recovery time of 40–45 days was observed.

Among children having had a diarrheal event, we observed a 10x higher relative abundance of *Prevotella*. After travelling, a higher abundance of two *Bacteroides* species and 40% less *Lachnospiraceae* were seen. Antibiotic use did not correlate with changes in the abundance of any bacteria.

**Conclusion:**

We present data showing that Danish children in daycare have stable intestinal microbiota, resilient to the exposures investigated. An early age-dependent increase in the diversity was demonstrated. Diarrheal episodes decreased alpha diversity with an estimated recovery time of 40–45 days.

**Electronic supplementary material:**

The online version of this article (10.1186/s12866-018-1367-5) contains supplementary material, which is available to authorized users.

## Background

The healthy human intestinal bacterial microbiota has been described as a diverse microbial community dominated by *Firmicutes*, *Bacteroidetes* and *Actinobacteria* and to a lesser extent *Proteobacteria* and *Verrucomicrobia* [1]. Various exposures, including foreign travel, consumption of antibiotics and environmental factors have been shown to greatly influence the structure of the intestinal microbiota [2–6]. Likewise, the composition and diversity of the human intestinal microbiota has been associated with various gastrointestinal diseases [7–11]. Taxa with a relatively high abundance such as *Ruminococcaceae, Enterobacteriaceae, Fusobacteriaceae, Pasteurellaceae* and *Veillonelleceae* have been associated with Crohn’s disease [7, 12] and *Desulfovibrio* with ulcerative colitis [13]. Meanwhile a ‘healthy microbiota’ has been associated with an increased abundance of *Faecalibacterium prautsnitzii*, *Escherichia coli*, *Oscillibacter* spp. and *Bifidobacterium* [14–17]*.* Furthermore, decreased bacterial alpha diversity has been associated with several gastrointestinal diseases [12, 18–20].

The emergence of new technologies has allowed for a deeper understanding of the interplay between bacterial communities in the gut, e.g., when challenged by diarrheal episodes and in the following period of recovery [14, 21, 22]. High-throughput 16S rRNA gene sequencing has provided an opportunity for a more holistic approach to monitoring microbial interactions in health and disease compared with conventional culturing of fecal samples, where the focus has been on identifying single enteric pathogens as causes of disease. By shifting the focus from individual bacterial species to studying entire microbial communities, it is possible to investigate and gain insight into how complex the alterations in the microbiota are in periods of health and disease.

Here, we present the first longitudinal study investigating compositional changes in the intestinal microbiota of children in daycare before and after diarrheal events and exposure to known risk factors (e.g., travel and use of antibiotics). Thus, we come a step closer to understand the dynamics of the intestinal microbiota in otherwise healthy children. In addition, we aim to define specific bacterial taxa that might confer an increased colonization resistance or greater resilience towards development of diarrhea.

## Results

We investigated the intestinal microbiome of 32 healthy children, with a median age of two years, for a period of one year. Our longitudinal observations enabled analysis of paired and non-paired data pertaining to the dynamics in the intestinal microbiota in Danish children in daycare.

### Sequencing depth

We created rarefaction curves for both observed richness and Shannon Diversity Index (H′) in order to determine the minimum sequencing depth that properly described the microbiota (Additional file 1: Figure S1). The observed richness was dependent on sequencing depth, but the rate of increase became significantly lower when including more than 2000 reads. The H′ reached a plateau when including more than 1000 reads. Consequently, we excluded 10 samples with less than 2000 reads for further analysis, and rarefied the remaining samples to 3500 reads per sample. This rarefied dataset was used for all analyses in the study.

### Stability of the microbiota

To identify the stability of the microbiota, we identified the core microbiota for each individual child (defined as the operational taxonomic units (OTUs) present in all samples from each individual child). The median relative abundance of the core microbiota in each sample was 95.9% (interquartile range (IQR): 92.6–97.4%, Additional file 2: Figure S2) and did not correlate with diarrheal episodes, antibiotic treatment, travelling (in or outside Scandinavia), or any combination of these perturbations.

### Beta diversity

**Fig. 1:**
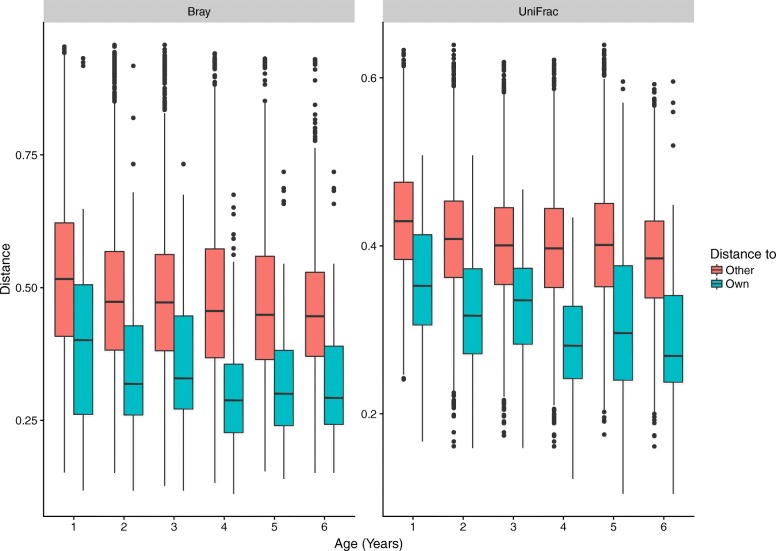
Boxplot showing intra- (green) and inter- (red) individual variation in gut microbiota over time according to age. The box-plot ranges from the 25th to the 75th percentile, with the 50th percentile represented by the black horizontal line

Using both Bray-Curtis and unweighted UniFrac distance metrics, we observed that samples differed more between children than within children (Fig. 1, Wilcoxon, *p* < 10^− 15^).

As verification of our findings, we used permutational multivariate analysis of variance using distance matrices (PERMANOVA) to determine the amount of the overall variation in beta diversity explained by the individual child. Based on Bray-Curtis distances and unweighted UniFrac distances the individual child explained 53.6 and 48.3% of the overall variation, respectively (*p* < 0.001).

The effect of age, travelling, diarrheal episodes, and antibiotic use on the microbial composition was tested using PERMANOVA, for both distance metrics (full results in Additional file 3: Table S1). The largest part of the variation was explained by the age of the child, 7.6% in the Bray-Curtis distances (*p* < 0.001). When testing the variation in the Bray-Curtis distances separately, travelling significantly explained 2.1% (*p* = 0.002), diarrhea 1.4% (*p* = 0.015), while antibiotic use did not significantly explain any of the overall variation (1.3%, *p* = 0.22). When all four variables were tested sequentially in one model (ordered as above), varying parts of the overall variation was explained by age (7.6%, *p* < 0.001), travelling (1.8%, *p* = 0.002), and diarrhea (1.2%, *p* = 0.014), whereas antibiotic use did not contribute significantly to explaining the variation observed in the samples. Furthermore, episodes of diarrhea explained additional 6.0% of the variation within each age group (*p* = 0.001).

For the unweighted UniFrac distances, the age of the child at sampling correspondingly explained the largest part of the variation (9.3%, *p* = 0.001). On the other hand, antibiotic use explained 2.1% (*p* = 0.001), while both travelling and diarrhea explained 1.4% (*p* = 0.002 and *p* = 0.001, respectively). When including all variables in the analysis (in the same order as above), antibiotics explained 1.2% (*p* = 0.003), travelling 1.4% (*p* = 0.001), and diarrhea 1.1% (*p* = 0.012) of the overall variation. Regarding variation within groups, travelling explained additional 3.7% of the overall variation within the age groups (*p* = 0.009) and 0.9% within the antibiotic treatments (*p* = 0.047). No other combination of variables significantly explained any of the remaining variation.

### Alpha diversity

**Fig. 2:**
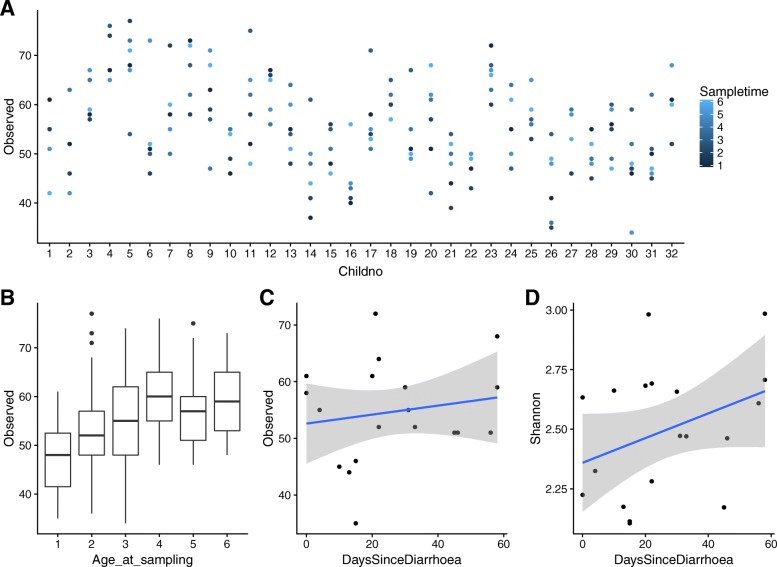
Alpha diversity. **a** Observed richness of each sample grouped for each child and colored by sampling time. **b** Boxplot of the observed richness grouped by the age at sampling. **c** The observed richness and D) H′ as function of days since last diarrheal episode (dots), with the blue line showing a glm and the grey area the 95% confidence interval

To test if the four variables (age, travelling, diarrhea, and antibiotic use) had an impact on the diversity of the individual samples, we investigated any correlation between the variables and the alpha diversity using both observed richness and H′. The observed richness of each sample indicated no temporal trend within each child (Fig. 2a). When grouping the samples by child age, we observed a significant correlation between age at sampling and observed richness (analysis of variance (ANOVA), *p* < 10^− 4^, Tukey Honest Significant Differences (TukeyHSD): 1 year lower than 2–6 years, *p* = 0–0.003, Fig. 2b), with a similar trend for H′ (ANOVA, p < 10^− 7^, TukeyHSD: 1 year lower than 3–6 year, *p* = 0.0003–0.037, data not shown). Furthermore, for children with a diarrheal event within the last 60 days, the alpha diversity could be explained as a function of the time passed after the reported diarrheal episode. To investigate this, we used generalized linear models (glm) (observed richness: glm, *p* < 10^− 10^, Fig. 2c. H′: glm, *p* < 10^− 13^, Fig. 2d). Comparing these models to the mean observed richness (55.76) and H′ (2.59) in samples where the child had not had a diarrheal events in the two months prior, the observed richness recovered after 39.86 days and H′ recovered after 44.75 days.

### Effects of diarrhea, travel and antibiotic use on single bacteria abundances

**Fig. 3:**
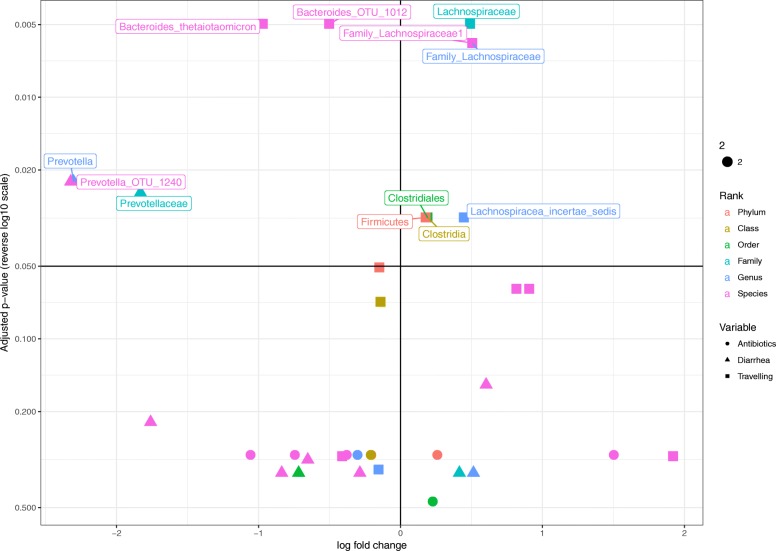
Vulcano plot of the differential abundant bacteria. X-axis reflects the log10 value of fold change in abundance for each variable (exposed no/yes). Y-axis is -log10 of the adjusted *p*-values. The shape indicates the exposure (Antibiotics: Circle, Diarrhea: Triangle, Travelling: Square) and the color indicates the taxonomic rank from phylum to species level

We used a permutational test to determine the significance of any differences in abundance of specific bacterial taxa following diarrheal episodes, travel, and antibiotic usage. We included all OTUs with a minimum average abundance of 0.05%, and performed the analysis at all taxonomical levels. The test was performed for diarrhea (23.8%, 41 of 172 samples [9 NA]), travelling (27.0%, 47 of 174, [7 NA]), and antibiotic use (7.0%, 12 of 172 [9 NA]). Across all taxonomic ranks we identified 51 (Antibiotic use: 14, Travelling: 18, Diarrhea: 19) association with *p* < 0.05, but only 12 (Travelling: 9, Diarrhea: 3) survived correction for false discovery rate (Fig. 3, Additional file 3: Table S2).

Children who had experienced at least one diarrheal episode within the last two months, had an approximately 10-fold increase of the genus *Prevotella* (2.8% vs. 0.28%, adjusted *p*-value = 0.01).

Children, who had travelled within a period of two months prior to sampling had an increased relative abundance of two *Bacteroides* species, *B. bacterium_NLAE-zl-P191* and *B. thetaiotaomicron*, (1.1% vs. 0.65 and 2.1% vs. 0.81%, respectively, adjusted *p*-values = 0.008). Furthermore, a decrease of the family *Lachnospiraceae* was observed (9.1% vs. 14.8%, adjusted *p*-value = 0.009).

Antibiotic usage did not cause any significant changes in relative abundances of the bacteria in our data set.

**Fig. 4:**
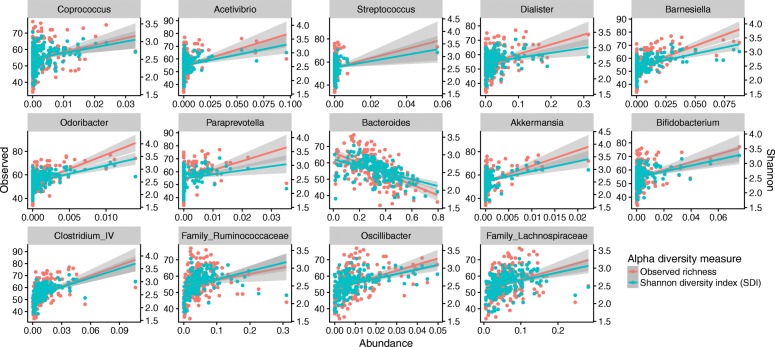
Correlation between alpha diversity and bacterial abundance. Alpha diversity plotted as a function of abundance in each sample, with observed richness (red) scale on the left y axis and H′ (blue) on the right y axis. The lines represent a general linear model with the grey area showing 95% confidence intervals

We tested if any genera, with a mean read count higher than 1 per sample, correlated with the alpha diversity. Using the DAtest function, we estimated the efficiency of all linear models (data not shown) and based on these data, we selected both linear regression modelling (false discovery rate: 0.056, true positive rate: 0.333) and log linear regression modelling (false discovery rate: 0.040, true positive rate: 0.200). Fourteen genera were determined to have a significant correlation with both H′ and observed richness, using both methods. The majority was observed to be positively correlated with alpha diversity (Fig. 4, Additional file 3: Table S3). The only negatively correlated genus was *Bacteroides*, which is the most abundant genus in this study (mean: 32.7%, IQR: 22.5–43.3%).

## Discussion

We found that children in daycare had a stable intestinal microbiota, resilient to the perturbations investigated. The core microbiota in each child dominated their microbiota, above 95% mean relative abundance, throughout the study; none of the tested exposures correlated with a decrease in the relative abundance of the core microbiota in general, nor when looking at each child separately (Additional file 2: Figure S2). The analysis of beta diversity variation showed a greater similarity between samples collected from the individual child compared with samples collected from other children (Fig. 1), which further highlights the considerable individual contribution to the unique composition of the microbiota even in small children. In addition, the individual child from which a sample was collected explained approximately half of all variation between samples. This was observed using both the Bray-Curtis distance metric, a merely abundance-based approach, and unweighted UniFrac distances, which is a distance metric based on the phylogenetic similarity between the bacteria present in the samples.

Age at sampling was a significant factor for the variation observed between samples; it explained additional 7.6–9.6% of the variation, supporting earlier studies, and showing how the intestinal microbiota is still undergoing some maturation at this young age [23–25]. Travel interestingly explained more of the variation, when using a purely abundance-based distance metric (2.1% by Bray-Curtis), as opposed to the comparison of the phylogenetic similarities between the bacteria present in the samples (1.4%, unweighted UniFrac). The difference in explained variance could indicate that microbiota changes associated with travel relates to a shift in the relative abundance of the bacteria present, or introduction of closely related bacteria, and to a lesser extent to introduction of foreign bacteria. Diarrheal events only explained 1.4% of the variation, using either distance metric, which was less than expected, when considering the association between stool consistency and microbiota composition [26]. Antibiotic usage explained more of the variation, when using unweighted UniFrac (2.1%) compared with Bray-Curtis (1.3%); it is interesting to note that only the presence/absence based unweighted UniFrac distance explained a significant variation from antibiotic usage. This difference in the amount of variation beta-diversity explained for the two distance metrics suggest that antibiotic use affects low-abundant bacteria, which might have been removed from the child’s microbiota, and to a lesser effect on the dominant bacteria. To speculate any further on the low variation explained by travelling, diarrhea, or antibiotic use should be subject to caution, due to the varying time intervals between antibiotic use and sample collection in our study. The fixed sampling interval meant that the time between diarrheal events and the sample collection ranged from 0 to 59 days. This large variation in the time passed since diarrheal event could explain this discrepancy, as it is difficult to differentiate between the direct impact of diarrheal events and the microbiota resilience, the ability of the microbiota to reestablish itself.

Looking at the alpha diversity characteristics, observed richness and H′, no time-dependent patterns within each child were observed. However, an overall increase in alpha diversity was observed in the younger children, from 1 to 3 years of age (Fig. 1a, b). In addition to the age-dependent trend in alpha diversity, we found a clear indication of alpha diversity being lower after a diarrheal event and that a time-dependent increase in alpha diversity related to the number of days since the diarrheal event took place; this development was clearest when analyzing H′ (Fig. 1c, d). When comparing alpha diversity/time correlation to samples without reports of diarrheal episodes, we could estimate that the alpha diversity reached the mean level of children without diarrheal episodes after 40–45 days. This dose-dependent effect in means of days past since a diarrheal event indicates a strong correlation between diarrhea and alpha diversity in the microbiota of small children.

In an effort to identify any more specific impact of travelling, diarrheal events and antibiotic use on the microbiota, we performed a permutation test to identify bacteria, with a different relative abundance between groups (Fig. 4). *Prevotella* was significantly increased following diarrheal events; we saw a 10x higher average relative abundance in children having had a diarrheal event. Such an increase does not necessarily implicate *Prevotella* as a causative agent in the diarrheal episode, but might suggest early re-colonization of *Prevotella* in the period of recovery after a perturbation of the microbiota caused by diarrhea. This is likely to be the first step in the succession of re-colonization events, and has also been described as one of the stages after *Vibrio cholera*-induced childhood diarrhea, where increased abundance of *Prevotella* is associated with late-stage recovery from infectious diarrhea and interpreted as a return to a healthy gut microbiota [27].

In children, who had been travelling, we saw an increase of two *Bacteroides* species, *B. thetaiotaomicron* (2.6 times higher) and *B. bacterium_NLAE-zl-P191* (1.7 times higher). In these children, we observed a 40% decrease in the family Lachnospiraceae, a member of the class *Clostridia*. In general, the phylum *Bacteroidetes* tended to be increased by 16% after travelling (not statistically significant), while the phylum *Firmicutes* was decreased by 16%. This change in bacterial abundance might be associated with changes in diet during travelling. No significantly differential abundant bacteria were seen in relation to antibiotic use.

Lastly, we looked at possible correlations between alpha diversity and abundance of individual bacterial genera. We found 13 genera positively correlated with alpha diversity, and only one (*Bacteroidetes*) was negatively correlated with alpha diversity (Fig. 4). That the most abundant genus in the entire dataset is negatively correlated with alpha diversity is not surprising, since a high relative abundance of a single OTU will decrease the number of other OTUs found within a given number of observations.

This longitudinal cohort study of healthy children aged 1–6 years with samples collected over a 12-month period enabled us to monitor the intestinal microbiota of children in daycare from a perspective never presented before. As a study based on the use of 16S rRNA gene amplicon sequencing the strengths and limitations of the technique is inherent in this study. The high sequencing depth and number of reads per sample give a very good foundation for the multiple statistical tests used, and, even more importantly, it allowed us to identify bacteria in different phases of the child’s life, through disease and health, which would be difficult to identify using a classical culture-dependent approach. Fecal culturing for microbial pathogens was performed and described earlier [16]. The findings reflected that the children were carriers of a numerous pathogens during the observation year. No correlations throughout the year for each child could be found by culturing.

The main limitation of this study is the level of resolution of the data, as we have amplified and sequenced the V4 region of the 16S rRNA gene followed by clustering of reads into OTUs with a 97% identity threshold. Therefore, many OTUs will contain multiple species, and even genera, as these may differ by less than 3% within the V4 region. Furthermore, as we cannot even differentiate species, this also means that differentiation between e.g.*,* a commensal *E. coli* and a pathogenic enterotoxin-producing *E. coli* was not possible*.* Our study design, with samples collected at fixed 2 month intervals, provided a unique opportunity to compare samples before and after perturbation of the microbiota. Lastly, any partial recovery of the gut microbiota occurring after any exposure will have obscured the effect of the exposure due to the variable intervals of time between sampling.

## Conclusion

We here present a study of the fecal microbiota of children in Danish daycare, observed over a 12-month period, and the impact of three types of exposures for each child: Travelling, diarrhea, and antibiotic use. The fecal microbiota of each child were dominated by a stable core microbiota, and the abundance of the core microbiota were not affected by the exposures investigated. While a few specific bacteria were affected by travelling and diarrhea. This indicate that already at this early age, contradictory to the common conception, the children have a very early establishment of a highly individualized and robust fecal microbiota community. We did observe an age-dependent increase in alpha diversity of the intestinal microbiota; this was seen strongest in the youngest children (1–3 years of age).

Most interestingly, our analysis of the alpha diversity, following diarrheal events, showed a clear decrease in alpha diversity and an estimated recovery period of 40–45 days.

## Methods

### Subjects and samples

A cohort of 179 healthy children aged 1 to 6 years was established to investigate the incidence of enteric pathogens in relation to diarrheal episodes in daycare in Denmark. The cohort were created to investigate incidence and clinical significance of Enteroaggregative *Escherichia coli* in healthy Danish children in daycare and the primary results, using conventional microbiological tests for enteric pathogens, have been published [16, 17, 28, 29].

**Fig. 5:**
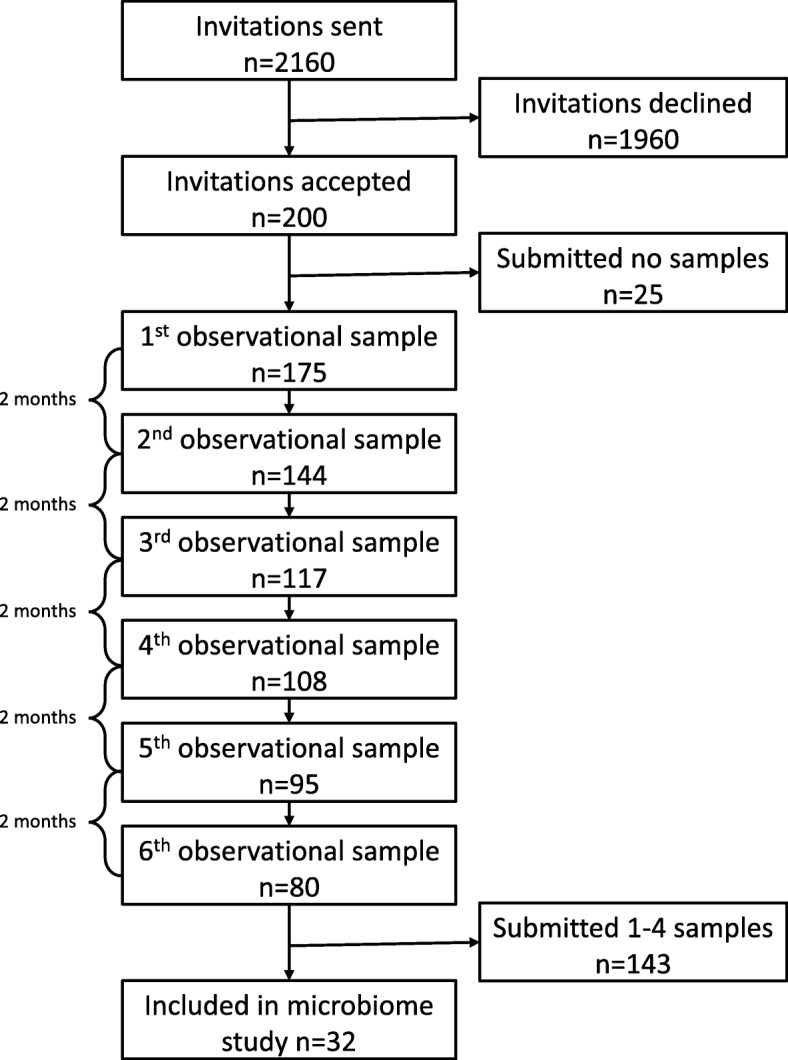
Flow chart of participating children. A cohort of healthy children aged 1 to 6 years was established. Each child was included for a one-year period with a follow-up point every second month with submission of a fecal sample and a questionnaire. In total, 2160 children were invited to participate in the cohort, 200 (9.6%) accepted the invitation, and of these 175 (87.5%) submitted at least one sample. Of these 32 (18.3%) submitted 5 (1) or 6 (31) fecal samples, and the samples (*n* = 191) from these children were analyzed using 16S rRNA gene amplicon sequencing

The study was designed as a dynamic cohort study, where each child was included for a one-year period with a follow-up point every second month with submission of a fecal sample and a questionnaire. The questionnaire inquired about gastrointestinal symptoms, use of antibiotics and various exposures in relation to gastrointestinal disease, and was completed by the parents. In total, 2160 children were invited to participate in the cohort, 200 (9.6%) accepted the invitation, and of these 175 (87.5%) submitted at least one sample. Of these 32 (18.3%) submitted 5 (1) or 6 (31) fecal samples, and the 191 samples from these children were analyzed using 16S rRNA gene amplicon sequencing (Fig. 5).

### DNA extraction

Fecal samples were collected by at home and shipped to Statens Serum Institut, Copenhagen. The fecal was transferred to Eppendorf® tubes and kept at − 80 °C. Bacterial DNA was extracted from fecal material using the Qiagen QIAmp DNA Stool Mini Kit (QIAGEN, Hilden, Germany) according to the manufacturer’s instructions. Briefly, 200 mg fecal material was mixed with 1.4 mL ASL buffer and vortexed until homogenization. In the next step, 0.2 g sterile zirconia/silica beads (diameter 0.01 mm, BioSpec Products ROTH Karlsruhe, Germany) were added, and samples were processed on the Tissuelyzer (Qiagen Retsch GmbH, Hannover, Germany) for 6 min at 30 Hz. This was followed by lysis for 5 min at 95 °C. The final sample preparation was eluted in 100 μL buffer [30–32].

### PCR and Miseq analysis

16S rRNA gene amplification was performed using a nested two-step protocol, targeting the hypervariable V4 region of the 16S rRNA gene. In the first step, the V3 and V4 regions were amplified using the modified broad-range primers Uni340F (5’ – CCT AYG GGR BGC ASC AG – 3′) and Uni806R (5’ – GGA CTA CNN GGG TAT CTA AT – 3′) [33–36]. Amplification was performed in 96-well microtiter plates with a reaction mixture consisting of 1X AccuPrime PCR Buffer II, 0.6 U AccuPrime Taq DNA Polymerase (Invitrogen, Life technologies, CA, US), 0.5 μM primer 341F, 0.5 μM primer 806R, and 2.0 μL template DNA, resulting in a total volume of 20.0 μL for each sample. The first PCR step was performed using the following program: 2 min at 94 °C (denaturation), 30 cycles of 20 s at 94 °C (denaturation), 30 s at 56 °C (annealing) and 40 s at 68 °C (elongation), and lastly 5 min at 68 °C (final extension). A negative template-free control and a positive control were applied for each plate containing 2.0 μL DNA from a known bacterial mock community (1.0 ng/μL; HM-782D, BEI Resources, VA, US). PCR products were quantified using the Quant-iT PicoGreen quantification system (Life Technologies, CA, US). Prior to further analysis, dilution of samples with a DNA concentration of more than 6.0 ng/μL was performed to achieve a concentration of approximately 3.0–6.0 ng/μL.

In the second PCR step, we targeted the V4 region while also adding sequencing primers and adaptors to the amplicon products. This step was performed as above, with 2.0 μL of the diluted amplicon products and sequence-specific primers, with sequencing adaptors and unique index combinations for each sample as described by Mortensen et al. 2016 [37]. In addition, the number of PCR cycles was reduced to 15. Amplicons were purified with Agencourt AMPure XP Beads (Beckman Coulter Genomics, MA, US) according to the manufacturer’s instructions using 0.7X volume beads and quantified as previously described. Equimolar amounts of the amplification products were pooled in a single tube. Pooled DNA samples were concentrated using the DNA Clean & ConcentratorTM-5 Kit (Zymo Research, Irvine, CA, US) according to the manufacturer’s instructions, and the final DNA concentration of the pooled library was determined using the Quant-iT™ High-Sensitivity DNA Assay Kit (Life Technologies) according to the instructions of the manufacturer. Amplicon sequencing was performed on the Illumina MiSeq Desktop Sequencer (Illumina Inc., CA, US). The samples were sequenced using the MiSeq Reagent Kits v2 (Illumina Inc., CA, US), and 5.0% PhiX was added as internal control. We performed 250 paired-end sequencing with dual-index reads.

For the development of OTU tables, taxonomic assignment, and phylogenetic analysis, we used our in-house bioinformatics pipeline [38]. Briefly, the pipeline used several tools to perform trimming (bioDSL [https://github.com/maasha/BioDSL]), mate pairing and quality filtering (usearch v7.0.1090 [39]), OTU clustering and singleton removal (97%, UPARSE [40]), chimera checking against the GOLD Database [41], taxonomical classification of representative sequences (Mothur v.1.25.0 [42]), construction of phylogenetic tree (PyNAST [43], FastTree [44], and filter_alignment.py [45]), and lastly, alignments against the 2011 version of Greengenes [46].

### Data analysis

Statistical analysis was performed in R (v3.4.1) [47], using several R packages. Import of the sequencing data (OTU table, taxonomic table, and phylogenetic tree) and sample information into R and general analysis were performed using the phyloseq package [48]. We used two measures of alpha diversity: observed richness (the number of unique OTUs found within each sample) and Shannon Diversity Index (H′) (a composite measure of the bacterial richness and the evenness of the relative bacterial abundance within each sample); both measures were calculated using phyloseq functionality. Using phyloseq functions we created rarefaction curves, determined a minimum sequencing depth, removed insufficiently sequenced samples and created a rarefied OTU table used for further analysis. For comparison of the overall compositional similarities and differences between the samples (beta diversity), we used two different distance metrics: Bray-Curtis similarity (based on relative abundances) and unweighted UniFrac distance (based on the phylogenetic differences between OTUs present or absent in the samples). In order to test how much of the overall variation in the microbial composition that could be explained by one or multiple categorical variables, PERMANOVA was performed using the function “adonis” from the R package Vegan [49]. Differences in alpha diversity between categorical variables were tested with ANOVA, followed by Tukey’s honest significant difference test (TukeyHSD) for between-group variance. Correlations with continuous variables were tested using generalized linear models (glm), all three using the R package stats. Differential abundance testing was performed using the test described by Thorsen et al. (2016) [50], the test perform between group comparison for all bacteria and includes false discovery rate (FDR) adjustment of *p*-values. Additional functions from the DAtest R package [51] were used to analyze correlations between the relative abundance of bacteria and alpha diversity. All plots were created using the R package ggplot2 [52].

## Additional files

Additional file 1: **Figure S1.** Rarefaction analysis of the species richness. Rarefaction curves showed a trend of an increasing diversity over time. Rarefaction curves were calculated for both observed richness and Shannon diversity index, grouped by time-point and with bars indicating standard deviations. The observed richness did not reach saturation before 20,000 reads, whereas the H′ reaches a maximum after 1000 reads (XLSX 20 kb)

Additional file 2: **Figure S2.** Abundance of Core microbiota in each sample, by sample number, grouped by child. Barplot showing the abundance of the core microbiota in each sample. The samples are grouped by which child they are from and ordered in chronological order. (PDF 41 kb)

Additional file 3: **Table S1.** Statistical output of all PERMANOVA analysis. **Table S2.** Table of bacteria, at all taxonomic levels, differential abundant with regard to exposures. Only taxa with an unadjusted *p*-value below 0.05 are included. **Table S3.** Table of genera being significantly correlated with both observed richness and Shannon diversity index. (PDF 70 kb)

## Abbreviations

ANOVA: Analysis of variance
glm: Generalized linear models
H′: Shannon diversity index
IQR: interquartile range
OTU: Operational taxonomic unit
PERMANOVA: Permutational multivariate analysis of variance using distance matrices
TukeyHSD: Tukey honest significant differences
