# Supplementary figures and images for: Stability and resilience of the intestinal microbiota in children in daycare – a 12 month cohort study
Source: BMC Microbiol. 2018 Dec 22;18:223. doi: 10.1186/s12866-018-1367-5 (PMC6303881; doi:10.1186/s12866-018-1367-5)

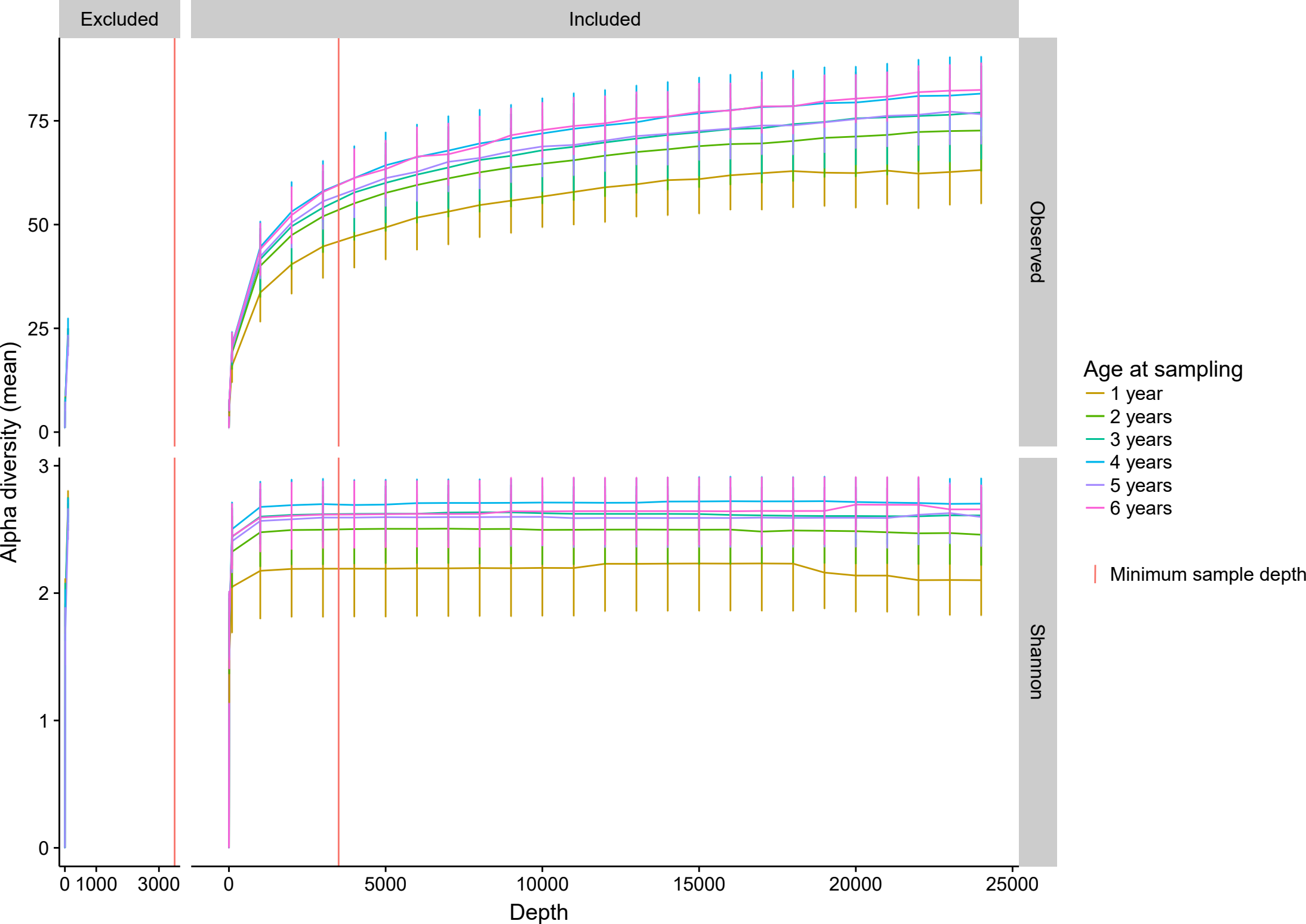

Supplement: Supplementary file 2 — Figure S2. Abundance of Core microbiota in each sample, by sample number, grouped by child. Barplot showing the abundance of the core microbiota in each sample. The samples are grouped by which child they are from and ordered in chronological order. (PDF 41 kb) [file 12866_2018_1367_MOESM2_ESM.pdf]

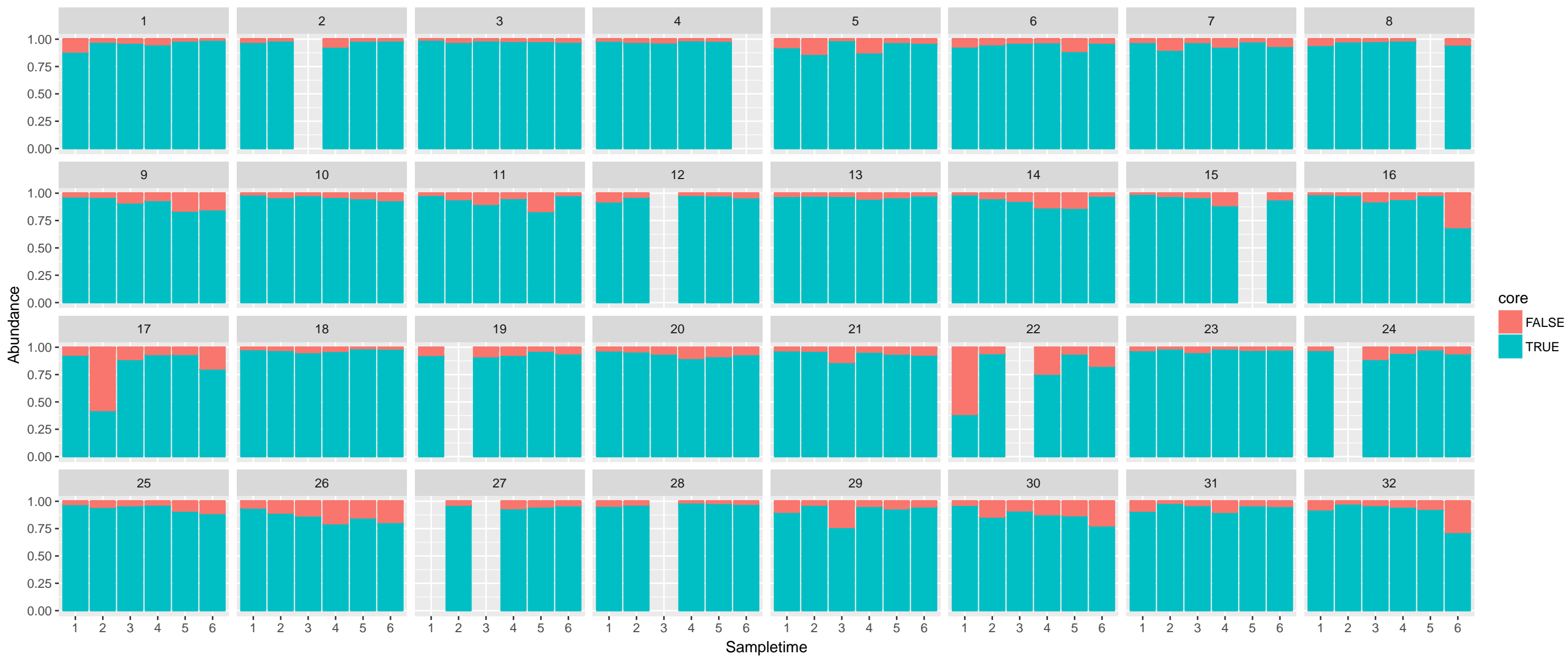

Supplement: Supplementary file 3 — Table S1. Statistical output of all PERMANOVA analysis. Table S2. Table of bacteria, at all taxonomic levels, differential abundant with regard to exposures. Only taxa with an unadjusted p-value below 0.05 are included. Table S3. Table of genera being significantly correlated with both observed richness and Shannon diversity index. (PDF 70 kb) [file 12866_2018_1367_MOESM3_ESM.pdf]
